# Supplementary material for: ProteinShader: illustrative rendering of macromolecules
Source: BMC Struct Biol. 2009 Mar 30;9:19. doi: 10.1186/1472-6807-9-19 (PMC2672931; doi:10.1186/1472-6807-9-19)
Supplement: Additional file 1 — ProteinShader program without source code. This compressed file contains the complete ProteinShader program including associated libraries, but no source code. A README.txt file gives an overview of the ProteinShader distribution, and the index.html file in the help subdirectory has directions on getting started with the program as well as a set of tutorials. [file 1472-6807-9-19-S1.zip › ProteinShader-beta-0_9_4-binary/help/api/org/proteinshader/structure/package-use.html]

Uses of Package org.proteinshader.structure (ProteinShader API)


|  |  |  |
| --- | --- | --- |
| **Overview** \| **Package** \| Class \| **Use** \| **Tree** \| **Deprecated** \| **Index** \| **Help** | |  |
| PREV   NEXT | **FRAMES**    **NO FRAMES**     **All Classes** |  |


---


## **Uses of Package org.proteinshader.structure**

| Packages that use org.proteinshader.structure | |
| --- | --- |
| **org.proteinshader.graphics** | Holds the drawing classes: Ribbon, Tube, FrenetFrames, Sphere, and Cylinder. |
| **org.proteinshader.graphics.adapter** | Holds the StructureToGraphics class, which is used to manage the use of the drawing classes and OpenGL display lists. |
| **org.proteinshader.graphics.displaylists** | Holds the classes needed to manage OpenGL display lists, which are used to cache reusable geometry for spheres, cylinders, ribbon segments, and tube segments. |
| **org.proteinshader.graphics.utils** | Holds a testing utility, class SegmentRenderer, which can be used for drawing ribbon and tube segments on-the-fly, rather than using cached geometry in the form of OpenGL display lists. |
| **org.proteinshader.gui** | Holds all of the Swing GUI components and their associated listeners, including class Renderer, which is registered as a listener for the GLCanvas object that is used a drawing surface. |
| **org.proteinshader.gui.components.controlpanel** | Holds the JPanels used in the right-side retractable control panel of the GUI. |
| **org.proteinshader.gui.viewing** | Holds important helper classes that the Renderer uses to control the view: Camera, Rotation, Lighting, *etc*. |
| **org.proteinshader.structure** | Holds the classes that store information from a Protein Data Bank file: Structure, Model, Chain, AminoAcid, Heterogen, Water, Atom, Bond, Helix, BetaStrand, Loop, *etc*. |
| **org.proteinshader.structure.factory** | Holds the factory classes needed for generating the Segment objects that hold the information needed for rendering ribbon and tube segments. |
| **org.proteinshader.structure.io** | Holds the classes needed for reading Protein Data Bank structure files. |
| **org.proteinshader.structure.io.demos** | Holds demo classes that were used for early testing of the io classes. |
| **org.proteinshader.structure.sort** | Holds the DrawableSorter class, which is needed for sorting Drawable objects based on their distance from the camera. |
| **org.proteinshader.structure.visitor** | Holds Visitor classes that know how to traverse the hierarchy of objects held by class Structure. |
| **org.proteinshader.structure.visitor.demos** | Holds demo classes that were used for early testing of the Visitor classes. |
| **org.proteinshader.structure.visitor.modifiers** | Holds helper classes for the ModifierVisitor class, which is used to modify Drawable objects (Atoms, Bonds, and Segments). |

Classes in org.proteinshader.structure used by org.proteinshader.graphics

****Segment****
  
          When a protein is drawn as a tube-like structure, a Segment is a
length of the tube that corresponds to an AminoAcid.

Classes in org.proteinshader.structure used by org.proteinshader.graphics.adapter

****Atom****
  
          Extends abstract class Drawable to add data that is unique to an atom
(atomID, type, temperature, occupancy, radius, *etc.*).

****Bond****
  
          Extends abstract class Drawable to add data that is unique to a bond
between two atoms.

****Drawable****
  
          This abstract class defines attributes and methods that are shared by
all drawable objects, including the xyz-coordinates for the Drawable's
center and methods that will allow each object to be sorted based on
distance from the camera.

****Model****
  
          A Model serves as a container for one or more Chains.

****Segment****
  
          When a protein is drawn as a tube-like structure, a Segment is a
length of the tube that corresponds to an AminoAcid.

Classes in org.proteinshader.structure used by org.proteinshader.graphics.displaylists

****Model****
  
          A Model serves as a container for one or more Chains.

****Segment****
  
          When a protein is drawn as a tube-like structure, a Segment is a
length of the tube that corresponds to an AminoAcid.

Classes in org.proteinshader.structure used by org.proteinshader.graphics.utils

****Segment****
  
          When a protein is drawn as a tube-like structure, a Segment is a
length of the tube that corresponds to an AminoAcid.

Classes in org.proteinshader.structure used by org.proteinshader.gui

****Atom****
  
          Extends abstract class Drawable to add data that is unique to an atom
(atomID, type, temperature, occupancy, radius, *etc.*).

****Chain****
  
          A Chain serves as a container for Residues (AminoAcid, Heterogen, and
Water) and Regions (Loop, Helix, and BetaStrand).

****Model****
  
          A Model serves as a container for one or more Chains.

****Residue****
  
          This abstract class serves as a container for one or more Atoms.

****Structure****
  
          Structure is the top-level container for objects that store
information on a protein structure from the
Protein Data Bank.

Classes in org.proteinshader.structure used by org.proteinshader.gui.components.controlpanel

****Atom****
  
          Extends abstract class Drawable to add data that is unique to an atom
(atomID, type, temperature, occupancy, radius, *etc.*).

****Chain****
  
          A Chain serves as a container for Residues (AminoAcid, Heterogen, and
Water) and Regions (Loop, Helix, and BetaStrand).

****Model****
  
          A Model serves as a container for one or more Chains.

****Residue****
  
          This abstract class serves as a container for one or more Atoms.

****Structure****
  
          Structure is the top-level container for objects that store
information on a protein structure from the
Protein Data Bank.

Classes in org.proteinshader.structure used by org.proteinshader.gui.viewing

****Drawable****
  
          This abstract class defines attributes and methods that are shared by
all drawable objects, including the xyz-coordinates for the Drawable's
center and methods that will allow each object to be sorted based on
distance from the camera.

Classes in org.proteinshader.structure used by org.proteinshader.structure

****AminoAcid****
  
          This concrete subclass of the abstract class Residue stores
information on an amino acid in a PDB structure entry.

****Atom****
  
          Extends abstract class Drawable to add data that is unique to an atom
(atomID, type, temperature, occupancy, radius, *etc.*).

****BetaStrand****
  
          This concrete subclass of Region holds information specific to a
protein BetaStrand.

****Bond****
  
          Extends abstract class Drawable to add data that is unique to a bond
between two atoms.

****Category****
  
          A Category object serves as a container for the Records in a PDB
structure entry.

****Chain****
  
          A Chain serves as a container for Residues (AminoAcid, Heterogen, and
Water) and Regions (Loop, Helix, and BetaStrand).

****Description****
  
          A Description object serves as a container for the Category objects
that hold Records describing a PDB structure entry.

****Drawable****
  
          This abstract class defines attributes and methods that are shared by
all drawable objects, including the xyz-coordinates for the Drawable's
center and methods that will allow each object to be sorted based on
distance from the camera.

****Helix****
  
          This concrete subclass of Region holds information specific to a
protein Helix.

****Heterogen****
  
          This concrete subclass of the abstract class Residue stores
information on a heterogen in a PDB structure entry.

****IDTest****
  
          This interface declares a processID() method that is intended to be
used as a helper method for constructors that need to perform tests
and/or any modifications on an ID.

****Loop****
  
          This concrete subclass of Region holds information on
the general "loop" regions found between well-defined
regions of secondary structure (helices and beta-strands).

****Model****
  
          A Model serves as a container for one or more Chains.

****Record****
  
          A Record object serves as a container for the lines of a PDB record.

****Region****
  
          Abstract class that contains references to a region of a Chain of
AminoAcids.

****Residue****
  
          This abstract class serves as a container for one or more Atoms.

****Segment****
  
          When a protein is drawn as a tube-like structure, a Segment is a
length of the tube that corresponds to an AminoAcid.

****Water****
  
          This concrete subclass of the abstract class Residue stores
information on a water molecule in a PDB structure entry.

Classes in org.proteinshader.structure used by org.proteinshader.structure.factory

****Region****
  
          Abstract class that contains references to a region of a Chain of
AminoAcids.

****Segment****
  
          When a protein is drawn as a tube-like structure, a Segment is a
length of the tube that corresponds to an AminoAcid.

Classes in org.proteinshader.structure used by org.proteinshader.structure.io

****Structure****
  
          Structure is the top-level container for objects that store
information on a protein structure from the
Protein Data Bank.

Classes in org.proteinshader.structure used by org.proteinshader.structure.io.demos

****Structure****
  
          Structure is the top-level container for objects that store
information on a protein structure from the
Protein Data Bank.

Classes in org.proteinshader.structure used by org.proteinshader.structure.sort

****Drawable****
  
          This abstract class defines attributes and methods that are shared by
all drawable objects, including the xyz-coordinates for the Drawable's
center and methods that will allow each object to be sorted based on
distance from the camera.

Classes in org.proteinshader.structure used by org.proteinshader.structure.visitor

****AminoAcid****
  
          This concrete subclass of the abstract class Residue stores
information on an amino acid in a PDB structure entry.

****Atom****
  
          Extends abstract class Drawable to add data that is unique to an atom
(atomID, type, temperature, occupancy, radius, *etc.*).

****BetaStrand****
  
          This concrete subclass of Region holds information specific to a
protein BetaStrand.

****Bond****
  
          Extends abstract class Drawable to add data that is unique to a bond
between two atoms.

****Chain****
  
          A Chain serves as a container for Residues (AminoAcid, Heterogen, and
Water) and Regions (Loop, Helix, and BetaStrand).

****Description****
  
          A Description object serves as a container for the Category objects
that hold Records describing a PDB structure entry.

****Drawable****
  
          This abstract class defines attributes and methods that are shared by
all drawable objects, including the xyz-coordinates for the Drawable's
center and methods that will allow each object to be sorted based on
distance from the camera.

****Helix****
  
          This concrete subclass of Region holds information specific to a
protein Helix.

****Heterogen****
  
          This concrete subclass of the abstract class Residue stores
information on a heterogen in a PDB structure entry.

****Loop****
  
          This concrete subclass of Region holds information on
the general "loop" regions found between well-defined
regions of secondary structure (helices and beta-strands).

****Model****
  
          A Model serves as a container for one or more Chains.

****Segment****
  
          When a protein is drawn as a tube-like structure, a Segment is a
length of the tube that corresponds to an AminoAcid.

****Structure****
  
          Structure is the top-level container for objects that store
information on a protein structure from the
Protein Data Bank.

****Water****
  
          This concrete subclass of the abstract class Residue stores
information on a water molecule in a PDB structure entry.

Classes in org.proteinshader.structure used by org.proteinshader.structure.visitor.demos

****Structure****
  
          Structure is the top-level container for objects that store
information on a protein structure from the
Protein Data Bank.

Classes in org.proteinshader.structure used by org.proteinshader.structure.visitor.modifiers

****Atom****
  
          Extends abstract class Drawable to add data that is unique to an atom
(atomID, type, temperature, occupancy, radius, *etc.*).

****Drawable****
  
          This abstract class defines attributes and methods that are shared by
all drawable objects, including the xyz-coordinates for the Drawable's
center and methods that will allow each object to be sorted based on
distance from the camera.

****Segment****
  
          When a protein is drawn as a tube-like structure, a Segment is a
length of the tube that corresponds to an AminoAcid.

---


|  |  |  |
| --- | --- | --- |
| **Overview** \| **Package** \| Class \| **Use** \| **Tree** \| **Deprecated** \| **Index** \| **Help** | |  |
| PREV   NEXT | **FRAMES**    **NO FRAMES**     **All Classes** |  |


---

# *Copyright © 2007-2008*
